# Case Report: Laser Ablation Guided by State of the Art Source Imaging Ends an Adolescent's 16-Year Quest for Seizure Freedom

**DOI:** 10.3389/fnhum.2022.826139

**Published:** 2022-01-25

**Authors:** Christos Papadelis, Shannon E. Conrad, Yanlong Song, Sabrina Shandley, Daniel Hansen, Madhan Bosemani, Saleem Malik, Cynthia Keator, M. Scott Perry

**Affiliations:** ^1^Jane and John Justin Neuroscience Center, Cook Children's Health Care System, Fort Worth, TX, United States; ^2^Department of Bioengineering, University of Texas at Arlington, Arlington, TX, United States; ^3^School of Medicine, Texas Christian University, University of North Texas Health Science Center, Fort Worth, TX, United States; ^4^Department of Radiology, Cook Children's Medical Center, Fort Worth, TX, United States

**Keywords:** epilepsy surgery, laser interstitial thermal therapy, source imaging, high-density EEG, magnetoencephalography

## Abstract

Epilepsy surgery is the most effective therapeutic approach for children with drug resistant epilepsy (DRE). Recent advances in neurosurgery, such as the Laser Interstitial Thermal Therapy (LITT), improved the safety and non-invasiveness of this method. Electric and magnetic source imaging (ESI/MSI) plays critical role in the delineation of the epileptogenic focus during the presurgical evaluation of children with DRE. Yet, they are currently underutilized even in tertiary epilepsy centers. Here, we present a case of an adolescent who suffered from DRE for 16 years and underwent surgery at Cook Children's Medical Center (CCMC). The patient was previously evaluated in a level 4 epilepsy center and treated with multiple antiseizure medications for several years. Presurgical evaluation at CCMC included long-term video electroencephalography (EEG), magnetoencephalography (MEG) with simultaneous conventional EEG (19 channels) and high-density EEG (256 channels) in two consecutive sessions, MRI, and fluorodeoxyglucose - positron emission tomography (FDG-PET). Video long-term EEG captured nine focal-onset clinical seizures with a maximal evolution over the right frontal/frontal midline areas. MRI was initially interpreted as non-lesional. FDG-PET revealed a small region of hypometabolism at the anterior right superior temporal gyrus. ESI and MSI performed with dipole clustering showed a tight cluster of dipoles in the right anterior insula. The patient underwent intracranial EEG which indicated the right anterior insular as seizure onset zone. Eventually LITT rendered the patient seizure free (Engel 1; 12 months after surgery). Retrospective analysis of ESI and MSI clustered dipoles found a mean distance of dipoles from the ablated volume ranging from 10 to 25 mm. Our findings highlight the importance of recent technological advances in the presurgical evaluation and surgical treatment of children with DRE, and the underutilization of epilepsy surgery in children with DRE.

## Introduction

For patients with drug resistant epilepsy (DRE), epilepsy surgery is the safest and most effective therapeutic approach to cure epilepsy (Ryvlin et al., 2014). Epilepsy surgery is particularly important in pediatric patients since it allows reversing psychological and social comorbidity responsible for dependence on family and society (Kossoff, 2011; Skirrow et al., 2011). Moreover, it offers the best opportunity to avoid a lifetime of disability, recover function, and improve the cognition and life quality of the patient (Reinholdson et al., 2015), since the pediatric brain possesses extensive neural plasticity (Gleissner et al., 2005).

The success of epilepsy surgery largely depends on the resection or disconnection of the area that is indispensable for generating seizures, called the epileptogenic zone (EZ) (Rosenow and Lüders, 2001). Early surgical evaluation strategies relied on semiology and the identification of seizure onset in the electroencephalography (EEG) signal. Currently, patients referred for epilepsy surgery undergo an extensive presurgical work-up, starting with magnetic resonance imaging (MRI) and EEG with synchronized video registration (video-EEG), and if needed fluorodeoxyglucose - positron emission tomography (FDG-PET) or ictal single-photon emission computed tomography (SPECT). This non-invasive phase is followed by invasive diagnostic techniques, such as the intracranial EEG (iEEG), which are regarded as gold standards for the recording of seizures and localization of the seizure onset zone (SOZ) that offers the best approximate of the EZ. Yet, they are costly; can be difficult due to the child's cooperation; require additional surgical intervention; and are limited to the area of electrode coverage. This makes it hard to judge whether the epileptogenic activity at the seizure onset really represents the seizure generator or is the result of propagation from other regions. Thus, the actual focus and extent of the seizure onset zone (SOZ) may be missed, leading to unsuccessful surgery.

Recent technological advances in the field dictate the integration of multiple levels of information derived from more sophisticated presurgical evaluation methods. In a recent review paper, Zijlmans et al. (2019) proposed the use of electric and magnetic source imaging (ESI/MSI) obtained from high-density EEG (HD-EEG) or magnetoencephalography (MEG) for almost all electroclinical syndromes. Their proposal was based on findings from several research groups, including ours, showing that ESI and MSI can provide valuable clinical information in the presurgical evaluation of patients with DRE, evaluated for surgical therapy (Mégevand et al., 2014; Papadelis et al., 2016; Tamilia et al., 2017, 2019, 2020, 2021; Plummer et al., 2019; Rampp et al., 2019; Ntolkeras et al., 2021; Papadelis and Perry, 2021; Ricci et al., 2021). ESI and MSI can provide a precise estimate of the irritative zone that can be interpreted as presurgical indicator of the EZ location (Tamilia et al., 2019). Thus, ESI and MSI solutions may obviate the need for invasive procedures or, in more complex clinical cases, can optimize their planning, and predict surgical outcome. However, these techniques are currently underutilized even in tertiary epilepsy centers.

Efficacy and safety of pediatric epilepsy surgery has been significantly improved over the last decades (Widjaja et al., 2011). Robotic assisted neurosurgery with interstitial thermal therapy (LITT) is increasingly utilized in recent years in the pediatric population, since it offers high degree of precision and a minimally invasive approach (Perry et al., 2017; Nelson et al., 2020). Such an approach is of particular interest in the pediatric population, as many brain pathologies in children present a considerable neurosurgical challenge. Normal brain anatomy can be compromised, and a child's developing brain is extremely vulnerable to injury (De Benedictis et al., 2017). Therefore, taking an accurate image-guided and minimally invasive approach to many pediatric neurosurgical procedures is highly desirable.

Progress in neuroimaging, electrophysiology, and minimally invasive surgical procedures has provided a comprehensive surgical management for children with DRE (Holthausen et al., 2013; Guan et al., 2016; Papadelis and Perry, 2021; Starnes et al., 2021). Yet, epilepsy surgery remains an underutilized treatment for children with DRE (Pestana Knight et al., 2015). In fact, only a third of pediatric surgical candidates proceed to surgery within 2 years of onset, despite this onset having occurred at <2 years of age in 60% of the children (Harvey et al., 2008; Ryvlin et al., 2014). Thus, many children with DRE continue treatment with antiseizure medications for several years before even considering epilepsy surgery as a treatment option. Here, we present a case of an adolescent female who suffered from DRE for 16 years, underwent LITT guided by ESI and MSI findings, and became seizure free (Engel 1; 12 months after surgery). Our findings highlight the importance of recent technological advances in the presurgical evaluation and surgical treatment of children with DRE, and the underutilization of epilepsy surgery in children with DRE.

## Case Description

### Patient's Demographics and Medical History

We studied a 17-year-old right-handed female with DRE who presented to Cook Children's comprehensive epilepsy monitoring unit for a second opinion. Her first seizure occurred at the age of 17 months of age, described as right eye and facial twitching with salivation. Her main seizure type at time of evaluation was described similarly, sitting up out of sleep with right eye and facial twitching, gurgling sounds, spitting, and yelling out with tensed, tremoring arms. These seizures occurred about four times per week. The patient's first neurology visit was at the age of 21 months, where she was referred due to her seizures. During neurological examination, reflexes, sensory exam, coordination, station, and gait were all unremarkable. The patient seemed playful with no evidence of cranial nerve abnormalities or nystagmus. There was no ankle clonus or increased reflexes. She had no history of febrile seizures or central nervous system infection but was hit in the head by a swing ~2 weeks prior to her initial seizure cluster. The impact knocked her to the ground, resulting in crying but no apparent loss of consciousness. As she grew, she continued to meet all developmental milestones until she was referred for evaluation of fine finger movement difficulties at the age of 7 years old. At age 11, she began having self-reported seizures with a sensation in the back of her throat followed by staring, which occurred both when the patient was awake and asleep. These continued to occur infrequently, about 3 or 4 times per month. Over the years, she tried multiple antiseizure medications including oxcarbazepine, levetiracetam, zonisamide, phenytoin, topiramate, lacosamide, valproate, benzodiazepines, in addition to trying the ketogenic diet. No reduction of severity or duration of seizures was observed following these treatments.

Prior evaluation at another level 4 epilepsy center suggested frontal lobe focus, favoring either midline or left lateral surface, with iEEG capturing several events that appeared to have bilateral ictal onset zones. MRI was reported as non-lesional. The recommendation was to place a deep brain stimulator.

### Presurgical Evaluation

The patient was admitted to Cook Children's Medical Center (CCMC) for second opinion. After reviewing the patient's medical record, a new phase one evaluation was performed. The patient received prolonged video-EEG monitoring, MEG with simultaneous conventional EEG (19 channels; conv-EEG), MRI (T1, T2, and FLAIR), and FDG-PET. After review of phase one data, the recommendation was to proceed with long-term iEEG with post-implant computerized tomography (CT) scan, and functional MRI for language localization.

### MEG/HD-EEG Recordings

**Figure 1:**
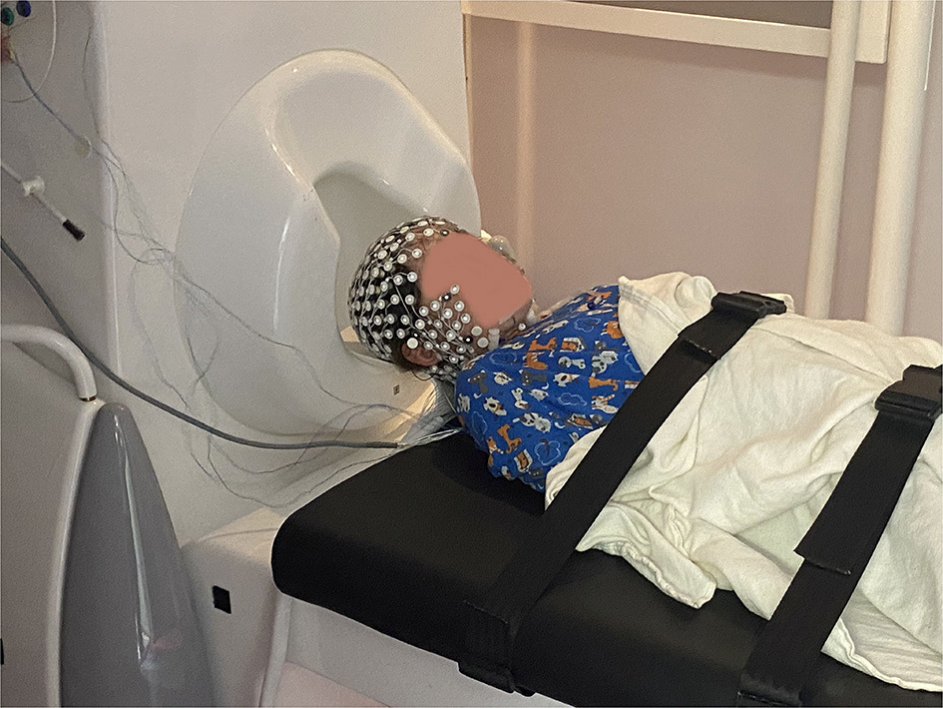
Setup of the MEG/HD-EEG recordings at CCMC. The patient lays down on a MEG-compatible bed, then the bed is pushed in to place her head inside the helmet. The HD-EEG recordings are performed simultaneously using a net that accommodates a high number of electrodes (i.e., 256). These electrodes do not require the application of gel in order to achieve high conductivity between the patient's scalp and the electrode, accelerating the patient's preparation time.

Simultaneous MEG with HD-EEG recordings were conducted as part of clinical work and research at the MEG facility of CCMC (Fort Worth, TX) across two separate days. The recordings were performed in a one-layer magnetically shielded room (Imedco, Hägendorf, Switzerland) with a whole-head Neuromag® Triux 306-sensor system (MEGIN, Finland). Conv-EEG leads were placed according to the international 10/20 system and connected to the MEG system for acquisition. HD-EEG was recorded using a MEG-compatible 256-channels Geodesic HD-EEG system (Magstim-EGI, OR, USA). The recordings were performed in a supine position following instructions to rest or sleep (Figure 1). Standard co-registration procedures were followed for MEG and HD-EEG according to the manufacturer's instructions.

### Identification of Interictal Epileptiform Discharges

Regardless of modality, data was filtered using band pass filter (1–70 Hz), notch filter (60 Hz and harmonics), and DC offset. For HD-EEG, electrodes located at the cheek and neck areas were removed (Vorderwülbecke et al., 2020). For MEG, the cardiac artifact was identified and removed from the signal using the Signal Space Projection technique (Uusitalo and Ilmoniemi, 1997). Interictal epileptiform discharges (IEDs) were marked by two independent reviewers who were blind to the patient's identity and to each other's markings.

### Source Localization of IEDs and Clusterness

Using Brainstorm software, the head points digitized prior to the recording were co-registered to the patient's MRI (Tadel et al., 2019). The patient's pre-operative MRI was used to create a realistic head model (Gramfort et al., 2010). For MRI segmentation, CAT12 from Brainstorm was used with default settings. For both ESI and MSI, a three-layer boundary elementary model (BEM) was used. The underlying generator of each IED was localized using Brainstorm's dipole scanning method (Tadel et al., 2011). The identified IEDs were localized independently based on modality, separate for conv-EEG, HD-EEG, MEG, and iEEG. The goodness-of-fit (GOF) was estimated for each dipole; only dipoles with GOF ≥ 50% were considered for further analysis. For each dipole, we calculated the number of dipoles, which are clustered around it, i.e., the number of dipoles that were located within a distance of 15 mm from it. Then, we normalized this measure by dividing it with its highest value for each modality (range: 0–100%). We will refer to such a normalized measure as the “clusterness” of the dipole (the higher the number, the more the dipole was surrounded by other dipoles). The clustering algorithm was developed in *Matlab*® (Math Works, MA) and is available for public use upon request (Ntolkeras et al., 2021).

### Source Localization of HFOs

Automatic detection of HFOs was performed using an in-house algorithm (Papadelis et al., 2016).

HFO source localization was performed using the wavelet Maximum Entropy on the Mean (wMEM) method (as proposed by von Ellenrieder et al., 2016), which is particularly well-suited to localize HFOs (Papadelis et al., 2016).

### Long-Term iEEG

Invasive recordings were performed with a 1,024 Hz sampling rate. A total of nine depth electrodes (8–16 linearly arranged contacts, 1.1 mm diameter, 3–5 inter-distance, AdTech, USA) were placed to cover the right temporal and frontal lobes guided by ROSA® (Medtech, Montpellier, France), with recordings collected across five consecutive days using XLTEK NeuroWorks (Natus Inc., USA).

### Electric Stimulation and Ablative Surgery With LITT

Based on the presurgical evaluation findings, the epilepsy team decided on ablation of the SOZ with LITT at the anterior right insula using two fibers for optimal coverage. Electrical stimulation of the areas to be ablated was initially performed to determine whether thermal ablation of the tissue would result in a deficit of eloquent functions (Hyslop and Duchowny, 2020). Using standard LITT procedures under real-time MR thermography, ablations were carried out along both laser fibers.

### Distance to Ablated Tissue and Surgical Outcome

For the purposes of this study, the ablated tissue was delineated in the patient's MRI formulating an ablated tissue volume. For each modality (i.e., conv-EEG, HD-EEG, and MEG), we estimated the mean Euclidian distance of dipoles with a clusterness of ≥75% from the closest point of the ablated volume. A board-certified pediatric epileptologist used the standardized Engel scale to evaluate the patient's post-surgical outcome from her most recent follow-up visit (Engel et al., 1993).

## Results

During the non-invasive phase of evaluation, video long-term EEG monitoring captured nine focal-onset clinical seizures, predominantly out of sleep, with maximal frontal onset. The seizures were characterized by a sense of fear, grabbing at the mouth and drooling, and tensing of the upper extremities. Electrographic runs of right frontal (i.e., F4 and F4/Fz) spike waves noted in the wake and sleep state without clinical correlation or evolution. MRI was initially interpreted as non-lesional. FDG-PET revealed a small region of apparent hypometabolism at the anterior right superior temporal gyrus. Functional MRI examination during verb generation and sentence completion demonstrated clusters of activation about the left inferior frontal region in keeping with the Broca area. Diffuse clusters of activation were also seen about the left posterior temporal region in keeping with the Wernicke area. No significant language-related activation was seen within the right cerebral hemisphere.

During the invasive phase of evaluation, iEEG captured one focal-onset clinical seizure that was characterized by high frequency onset activity (~60–90 Hz) in the right anterior insular lead more so than the lead immediately posterior in the mid right insula. The patient's seizures were characterized as focal onset aware seizures per ILAE classification. Electrical stimulation was provided to the insular depth electrodes revealed clinical sensation of arm tingling posteriorly. Typical clinical aura in the middle and anterior insula was duplicated with progressively lower stimulation as the onset zone in the anterior insula was neared.

**Figure 2:**
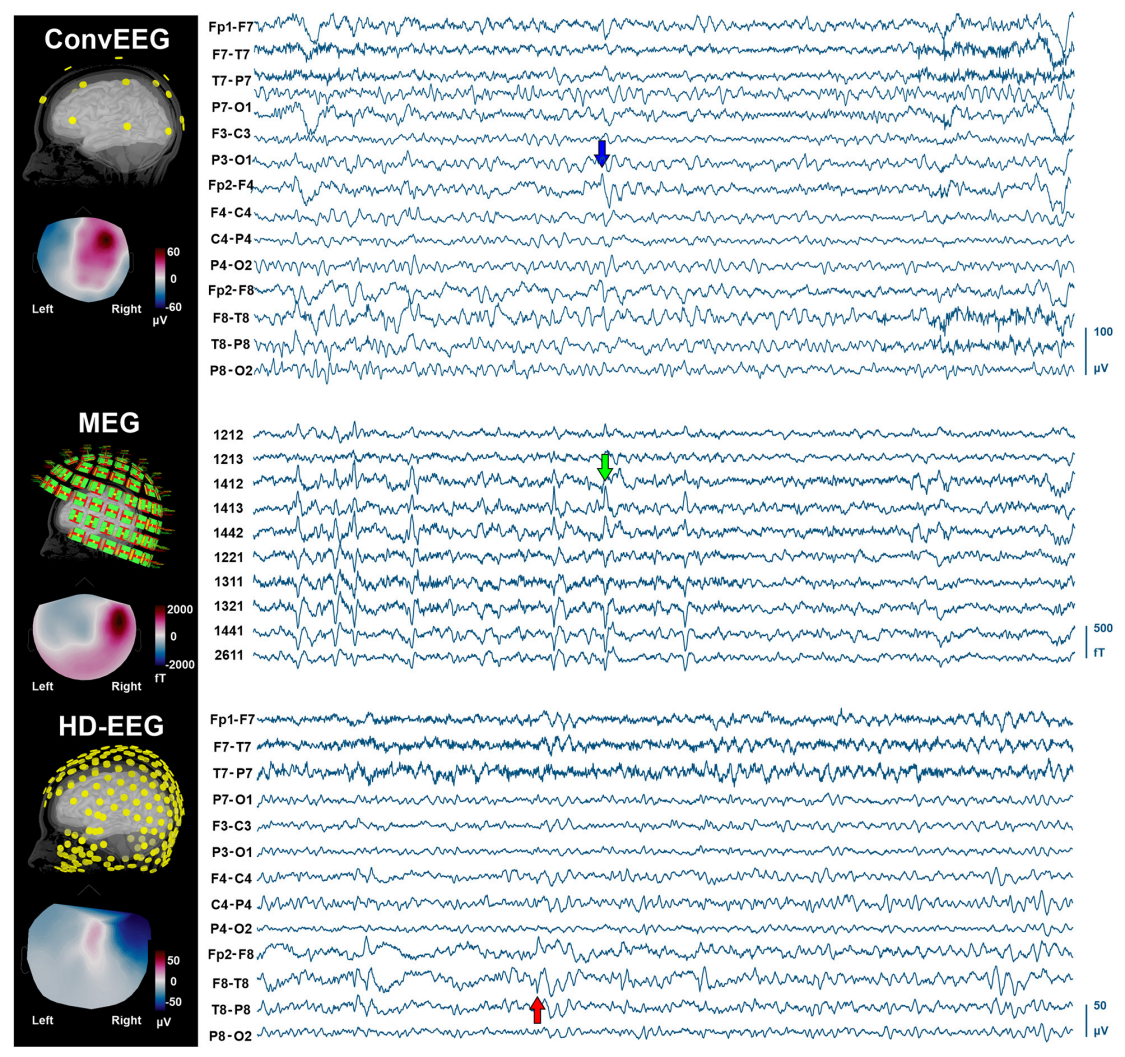
IEDs on non-invasive neuroimaging modalities (conv-EEG, MEG, and HD-EEG). (Left) Location of sensors for each modality co-registered with the patient's cortical surface reconstructed from the intra-operative MRI. Topographic maps at the peak of IEDs for each modality. (Right) Time-traces (10 s) with frequent IEDs for all three non-invasive modalities. Displayed time-traces of MEG and conv-EEG are synchronous.

**Figure 3:**
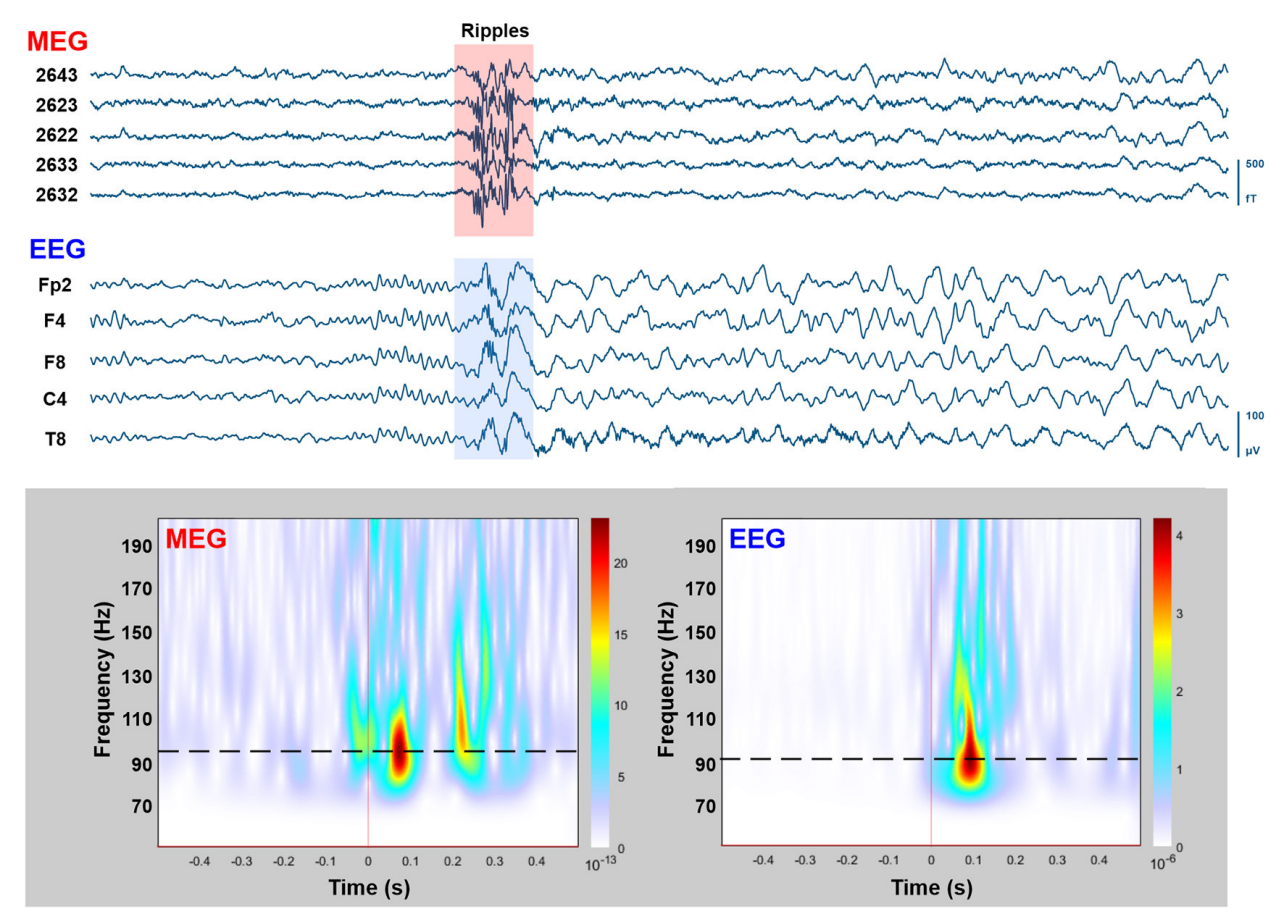
MEG/EEG recordings with HFOs overlapping on IEDs. Upper: Time-traces (10 s) of simultaneously recorded MEG (five planar gradiometers) and conv-EEG (five monopolar montage electrodes) recordings. Segment with HFOs in the ripple frequency band (80–200 Hz) overlaid on IEDs is highlighted. Lower: Time-frequency analysis (Morlet wavelets; 50–200 Hz) for the highlighted MEG and EEG segments (1 s duration). Peak frequency of ripple-event is displayed on time-frequency plots with dashed-lines.

During the simultaneous MEG with conv-EEG and HD-EEG recordings, we observed 54 IEDs on conv-EEG (11.87 spikes/min); 40 IEDs on HD-EEG (8.55 spikes/min); and 69 IEDs on MEG (17.38 spikes/min). Conv-EEG and HD-EEG showed frequent IEDs on electrodes Fp2-F4, F4-C4, Fp2-F8, and F8-T8 (blue and red arrows on Figure 2, respectively). Topographic mappings at the peak of IEDs for the conv-EEG and HD-EEG indicated an underlying epileptogenic focus in the right fronto-temporal areas. MEG detected frequent IEDs on sensors covering the right frontal and temporal brain areas (green arrow on Figure 2). The HFO-detector identified a ripple in both MEG and EEG showing maximum activity at sensors covering the right fronto-temporal brain regions (Figure 3—upper panel). The ripple had a peak activity at ~96 Hz for MEG and ~92 Hz for the EEG (Figure 3—lower panel). On iEEG, we identified 73 IEDs (14.81 spikes/min) mostly at electrodes placed at the right anterior insula.

**Figure 4:**
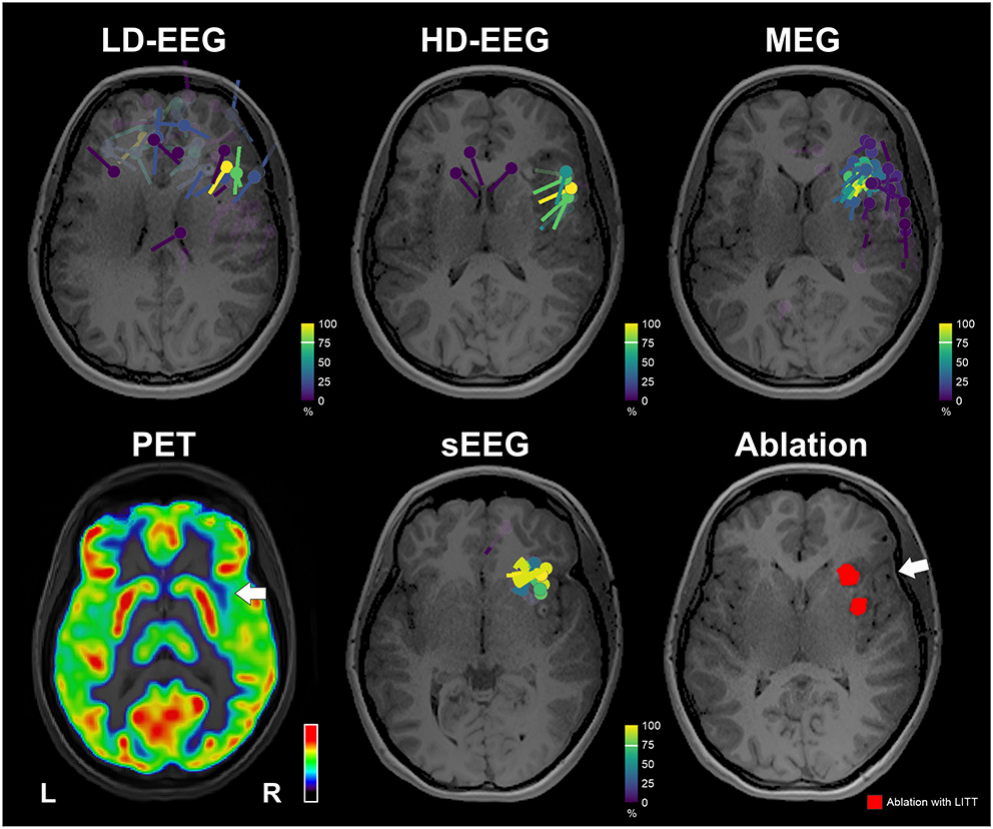
Neuroimaging findings with respect to ablation. Upper: Dipoles clustering overlaid on patient's MRI for the conv-EEG, HD-EEG, and MEG. Dipoles are color-coded based on clusterness [dipoles with high clusterness (have several other dipoles in its vicinity) are depicted with yellow; dipoles with low clusterness (few or no other dipoles in its vicinity) are depicted with magenta]. Lower: FDG-PET image shows a small region of hypometabolism at the anterior right superior temporal gyrus, indicated by white arrow. Dipoles after clustering for iEEG. Ablation volume (red) overlaid on patient's MRI. White arrow indicates the location of a possible focus of gliosis identified on patient's MRI. MRI and PET are displayed on neurological orientation (left to left).

The clustered dipoles showed a mean distance of 10.09 mm (±3.89) from the ablated tissue for the conv-EEG; 12.69 mm (± 2.50) for the MEG; 15.29 mm (±4.94) for the HD-EEG; and 9.18 mm (±1.84) for the iEEG (Figure 4). Localization of HFO with wMEM showed a peak activity in a distance of 16.32 mm from the right middle insula.

The ESI/MSI findings directed the epilepsy team and neuroradiologist to re-evaluate the patient's MRI focusing on the right anterior insula. Re-evaluation of T2 and FLAIR showed a mild hyperintense signal in the right frontal white matter extending between the cortex and frontal horn that seemed to represent a focus of gliosis or an abnormal myelination tract (white arrow in Figure 4).

The patient remains seizure free 12 months after surgery (Engel score: 1) receiving one antiseizure medication compared to three before surgery.

## Discussion

In this illustrative case, we localized predominant epileptiform activity in an adolescent who suffered from DRE for 16 years using ESI and MSI. A dipole clustering method, which our team developed (Ntolkeras et al., 2021), consistently localized the epileptiform activity in the right anterior insula. Data from different electrophysiological modalities (i.e., conv-EEG, HD-EEG, and MEG), which were analyzed independently to each other, showed congruent localization findings.

Subsequent review of the MRI with the coregistered ESI/MSI findings demonstrated a region of interest within the anterior right insula that appeared to represent focal cortical dysplasia. Based on these converging findings, the patient was referred for long-term iEEG monitoring with an implantation of several electrodes covering the right frontal and temporal areas, and the subsequent ablation of the epileptogenic focus with LITT, which rendered the patient seizure free (Engel 1).

Despite an extensive literature showing that ESI/MSI can assess the irritative zone of patients with DRE with good precision and in some cases predict surgical outcome (e.g., Nakasato et al., 1995; Knowlton, 2006; Mégevand et al., 2014; Englot et al., 2015; Rampp et al., 2019; Tamilia et al., 2019), these methods have gained only partial acceptance in clinical setting. A large consortium study of 25 epilepsy centers in Europe showed that less than half of these centers used these methods for presurgical evaluation (Mouthaan et al., 2016). ESI is based mostly on recordings of electric potential field using HD-EEG that makes use of a higher spatial sampling of scalp electrodes (at least 64 electrodes) compared to the standard 10–20 low-density EEG montage. It seems to yield more accurate localization results than the conv-EEG in comparative studies (Lantz et al., 2003; Brodbeck et al., 2011; Sohrabpour et al., 2015; Tamilia et al., 2019), although this finding could not be confirmed in a recent meta-analysis (Sharma et al., 2019). A retrospective study of 60 patients found that interictal ESI is superior to FDG-PET in 65%, and comparable to ictal single-photon emission computed tomography (SPECT) in 68% of cases (Russo et al., 2016). Here, we recorded HD-EEG with the highest spatial sampling that is currently available in the market (i.e., 256 electrodes). With the HD-EEG, we obtained an accuracy of ~15 mm in the localization of the EZ, which was here defined as the volume of ablated tissue. This is within the range of localization accuracy reported previously (Tamilia et al., 2019; Ntolkeras et al., 2021).

Previous studies from our group and others have showed that ictal and interictal ESI based on conv-EEG has significant clinical utility and can precisely localize the EZ (Coito et al., 2019; Tamilia et al., 2019; Ricci et al., 2021; Thurairajah et al., 2021). Our current case study is in line with these previous findings and highlight the role of conv-EEG in the presurgical evaluation of patients with DRE. In this case, we surprisingly found that conv-EEG offered a comparable localization accuracy of the epileptogenic focus with the HD-EEG. This may be explained by the use of our clustering approach, which seems to provide improved localization findings (compared to the traditional dipole fitting) in the presence of several IEDs (Ntolkeras et al., 2021).

MEG provides complimentary information to the EEG; approximately half of patients with epilepsy who do not show IEDs on scalp EEG present spikes on MEG (Knake et al., 2006; Heers et al., 2010; Kharkar and Knowlton, 2015). In our case, MEG and EEG showed a similar rate of IEDs. MSI with our clustering algorithm localized the EZ with an accuracy of ~12 mm. Such a localization is in line with our previous findings (Tamilia et al., 2019), and better than the localization of HD-EEG (~15 mm). Clustering of dipoles improved the localization of all modalities (ESI with conv-EEG; ESI with HD-EEG; and MSI with MEG) by several mm, similarly to our previous studies (Ntolkeras et al., 2021), and highlight the need for widespread use of more advanced MSI/ESI source localization algorithms (Pellegrino et al., 2020). Yet, a direct comparison of source localization abilities between the different techniques should consider the subjectivity of data review without considering a fix set of criteria (Kural et al., 2020) and the fact that HD-EEG and conv-EEG recordings were performed in different days.

This patient also benefited from two novel methodological approaches: the non-invasive localization of HFOs and the ESI performed on iEEG recordings. Among others, our group has shown that HFOs are promising interictal biomarkers of epilepsy, which can be recorded non-invasively with scalp EEG and MEG (Papadelis et al., 2009, 2016; Tamilia et al., 2017; Zijlmans et al., 2017; Thomschewski et al., 2019; Thomschewski et al., 2021). Surgical resection of the non-invasively identified HFO-zone predicts surgical outcome (Tamilia et al., 2021). Here, we localized an incident of HFO-activity observed in both conv-EEG and MEG and localized with MSI. In line with our previous findings, the HFO-zone was localized in close proximity to the EZ. The presurgical evaluation was completed with ESI performed on iEEG data. The iEEG recordings have limited spatial sampling, which may mislead the localization of the epileptogenic focus since the location of the recording contacts may differ from the actual source of the recorded activity (Ramantani et al., 2013). ESI performed on iEEG may overcome the limitations of the traditional iEEG interpretation and improve the delineation of the epileptogenic focus for epilepsy surgery (Alhilani et al., 2020). Indeed, among all localization techniques used in this case, the ESI performed on iEEG presented the lowest distance to the ablated volume (~9 mm). This could be partly explained by the fact that the delineation of the ablated tissue was mostly based on the interpretation of ictal iEEG data. Yet, ESI on iEEG data was performed retrospectively on interictal data and was blind to the clinical interpretation of ictal iEEG recordings.

The present study is an illustrative case of an extensive workup of a non-trivial patient who suffered from refractory epilepsy for 16 years. By use of an elegant combination of multiple new and/or underused technologies with tried and proven cornerstones of presurgical evaluation the patient became seizure free. Our study highlights the clinical value of multimodal neuroimaging, particularly interictal ESI/MSI, in the presurgical evaluation of children with DRE. It also demonstrates that although pediatric epilepsy surgery numbers have been increased in the past decade, epilepsy surgery remains an underutilized treatment for children with epilepsy. Despite an extensive literature demonstrating the significant clinical utility of ESI and MSI in the presurgical evaluation of patients with DRE, few tertiary centers use these methods in a systematic and comprehensive way; this is mostly due to lack of technical expertise to analyze and interpret these data and findings (Papadelis and Perry, 2021). Finally, this contribution is not limited to the use of highly sophisticated techniques, such as HD-EEG or MEG, but also when ESI is performed on conv-EEG data, typically available in all epilepsy centers. As more cases like this one will be presented (e.g., Hunold et al., 2014), we expect that the use of MSI/ESI in clinical epilepsy practice will accelerate during the next several years as new more advanced source localization methods and biomarkers are introduced.

## Data Availability Statement

The raw data supporting the conclusions of this article will be made available by the authors, without undue reservation.

## Ethics Statement

The studies involving human participants were reviewed and approved by Cook Children's IRB. Written informed consent to participate in this study was provided by the participants' legal guardian/next of kin. Written informed consent was obtained from the minor(s)' legal guardian/next of kin for the publication of any potentially identifiable images or data included in this article.

## Author Contributions

CP contributed in the concept of the experimental setup, the data analysis and interpretation, and the manuscript preparation. SC contributed in data analysis and manuscript preparation. YS and SS contributed in data collection. DH, MB, CK, and SM contributed in data interpretation and manuscript preparation. All authors contributed to the article and approved the submitted version.

## Funding

This study was supported by the National Institute of Neurological Disorders & Stroke (RO1NS104116-01A1; PI: CP; and R21NS101373-01A1; PIs: CP and S. Stufflebeam).

## Conflict of Interest

The authors declare that the research was conducted in the absence of any commercial or financial relationships that could be construed as a potential conflict of interest.

## Publisher's Note

All claims expressed in this article are solely those of the authors and do not necessarily represent those of their affiliated organizations, or those of the publisher, the editors and the reviewers. Any product that may be evaluated in this article, or claim that may be made by its manufacturer, is not guaranteed or endorsed by the publisher.
